# MiREx: mRNA levels prediction from gene sequence and miRNA target knowledge

**DOI:** 10.1186/s12859-023-05560-1

**Published:** 2023-11-22

**Authors:** Elena Pianfetti, Marta Lovino, Elisa Ficarra, Loredana Martignetti

**Affiliations:** 1 Department of Engineering, University of Modena and Reggio Emilia, Via Vivarelli 10/1, Modena, 41225 Italy; 2 Institut Curie, Rue d’Ulm 26, Paris, 75005 France; 3 Inserm U900, Paris, France; 4 CBIO-Centre for Computational Biology, Paris, France; 5 PSL Research University, Paris, France

**Keywords:** CNN, DNA, Gene expression levels, MiRNAs, Promoter, Sequence

## Abstract

Messenger RNA (mRNA) has an essential role in the protein production process. Predicting mRNA expression levels accurately is crucial for understanding gene regulation, and various models (statistical and neural network-based) have been developed for this purpose. A few models predict mRNA expression levels from the DNA sequence, exploiting the DNA sequence and gene features (e.g., number of exons/introns, gene length). Other models include information about long-range interaction molecules (i.e., enhancers/silencers) and transcriptional regulators as predictive features, such as transcription factors (TFs) and small RNAs (e.g., microRNAs - miRNAs). Recently, a convolutional neural network (CNN) model, called Xpresso, has been proposed for mRNA expression level prediction leveraging the promoter sequence and mRNAs’ half-life features (gene features). To push forward the mRNA level prediction, we present miREx, a CNN-based tool that includes information about miRNA targets and expression levels in the model. Indeed, each miRNA can target specific genes, and the model exploits this information to guide the learning process. In detail, not all miRNAs are included, only a selected subset with the highest impact on the model. MiREx has been evaluated on four cancer primary sites from the genomics data commons (GDC) database: lung, kidney, breast, and corpus uteri. Results show that mRNA level prediction benefits from selected miRNA targets and expression information. Future model developments could include other transcriptional regulators or be trained with proteomics data to infer protein levels.

## Introduction

Proteins enable the proper functioning of human organisms by regulating their biological processes and enabling cells to react in response to external and internal stimuli. Proteins are produced in ribosomes during the translation process from mRNA molecules.

The quantity of circulating mRNA (mRNA expression level) is crucial in understanding transcription and translation processes, as according to Liu and Vogel [1, 2] it is responsible for 40–50% of the variability in protein levels.

Organisms store the information that regulates their biological processes in DNA, which can be sequenced and written as a sequence of four letters representing the four amino acids (ACGT, Adenine, Cytosine, Guanine, and Thymine, respectively). However, the mechanism from DNA sequence to mRNA levels is not straightforward, and many processes are still unknown.

Post-transcriptional regulators are known to regulate the expression levels through covalent or structural mRNA modifications. Among post-transcriptional regulators, miRNAs are small RNA molecules, usually 22 bp long [3], which can repress the mRNA translation by binding to target mRNAs.

Depending on the extent of the mRNA-miRNA binding site, different repression mechanisms can occur:

- slicing of the mRNA,
- shortening of the poly-A cap of the mRNA, leading to the degradation of the mRNA,
- inhibition of mRNA translation, making it less efficient.

To the best of our knowledge, while some state-of-the-art (SOTA) works investigate the miRNA regulatory effect, none currently incorporate miRNA expression levels in predicting mRNA levels from the DNA sequence.

In this work we present miREx, a Convolutional Neural Network (CNN) model for predicting mRNA expression levels from gene sequence and miRNA post-transcriptional information. miREx’s architecture is inspired by Xpresso [4], a SOTA model for mRNA level prediction that exploits DNA sequence and gene features (e.g., number of exons/introns, gene length).

miREx predicts mRNA levels in four primary sites from The Cancer Genome Atlas (TCGA), namely lung, kidney, breast, and corpus uteri. The results show that by including selected miRNA expression levels, the model reaches higher performances.

## Related works

The ability to predict mRNA expression levels is fundamental for understanding the transcription process and the roles different regulatory molecules play. Indeed, some models leverage gene expression levels for patient stratification [5] and for predicting mRNA expression levels using other features instead of the DNA sequence. Commonly used features are TFs [6–8], chromatin features [9, 10], histone modifications [11], or their combination [12, 13]. These models usually outperform methods that use only the DNA sequence (like [14, 15]).

Concerning models that use the DNA sequence only, the ones with better performances exploit deep learning architectures. For instance, previous studies including Xpresso [4, 16, 17] use convolutional architectures, while models including Enformer [18, 19] use Transformers.

Indeed, Enformer is a state-of-the-art (SOTA) model with a transformer architecture. One of its inherent limitations is the challenge of incorporating information beyond the sequence it was designed to process. This limitation stems from the sequential nature of transformers, which excel at capturing long-range dependencies within sequences but are less accomodating for integrating additional contextual information. In contrast, models with convolutional architectures are more flexible in this regard. CNNs are well-suited for handling multi-channel inputs and can incorporate external information to enhance their performance. Indeed, recently, CNNs have been exploited in many medical and biological tasks [20–22].

The CNN-based SOTA model for mRNA level prediction from DNA sequence is Xpresso, that receives two inputs: the DNA sequence surrounding the Transcription Start Site (TSS) and the mRNA half-life features associated with each gene. The mRNA degradation rate impacts the steady-state mRNA levels [23], and the half-lives of mRNA molecules are one way to consider this process in the model.

It was shown in [24, 25] that features linked to mRNA half-life encompass specific aspects of gene structure. These include the length and CG content of particular gene regions, such as the 5’ UnTranslated Region (UTR), Open Reading Frame (ORF), and 3’ UTR, intron length and exon junction. These eight half-life features are considered in the Xpresso and miREx models.

## Materials and methods

In the following sections, we provide a comprehensive description of our methodology, encompassing a description of the data, the processing, and the architecture used.

### Data description

In this section, we provide an overview of the different data used by the model, along with references to sources for data acquisition. miREx predicts mRNA expression levels by exploiting a portion of the gene DNA sequence, mRNA half-life features, and miRNA targets.

#### Sequences and half-life features

Sequences and mRNA half-life features were obtained from [4]. A critical role in the transcription process is played by the promoter, a region of the DNA sequence, usually located upstream of the TSS [26] and spanning a few thousand base pairs. In addition, other regulatory sequences around the promoters have an essential role in transcription. Therefore, Xpresso and miREx models use a sequence centered on the TSS, thus including the promoter and parts of other regulatory elements. The sequence used has a length of 10,500 bases, 3000 preceding the TSS and 7500 following it.

#### miRNA targeting

TargetScan [27] is a state-of-the-art database for miRNA annotation and target description. Cumulative Weighted Context++ Scores (CWCS) from TargetScan were downloaded to embed miRNA target-specific information in the model. This score is a metric to assess the likelihood of a given miRNA binding to a target mRNA. Consequently, for each miRNA-gene pair, we have a CWCS. Therefore, our model also considers the effectiveness of miRNAs in the repression of gene expression.

#### mRNA and miRNA expression values

mRNA and miRNA data are obtained from the Genomic Data Commons (GDC) portal [28] for four primary sites: lung, kidney, breast, and corpus uteri. Each primary site can contain data from different cancer subtypes.

**Lung** Data from LUng ADenocarcinoma (LUAD) and LUng Squamous Cell carcinoma (LUSC) was downloaded. Mesothelioma (MESO) data was discarded since miRNA expression levels were unavailable. The dataset is composed of 982 samples (507 for LUAD and 475 for LUSC).

**Kidney** Data from KIdney CHromophobe (KICH), KIdney Renal Papillary cell carcinoma (KIRP), and KIdney Renal Clear cell carcinoma (KIRC) was downloaded. Sarcoma (SARC) was excluded due to the low number of samples. The dataset is composed of 871 samples (66 for KICH, 290 for KIRP, and 515 for KIRC).

**Breast** Data from Breast Invasive Carcinoma (BRCA) was downloaded. Diffuse Large B-Cell Lymphoma (DLBC) was excluded due to the low number of samples. The dataset is composed of 1076 BRCA samples.

**Corpus Uteri** Data from Uterine Corpus Endometrial Carcinoma (UCEC) was downloaded. Sarcoma (SARC) was excluded due to the low number of samples. The dataset is composed of 536 UCEC samples.

### Data processing

In each of the four datasets acquired, we preserved only those samples that included mRNA and miRNA expression values.

To make a fair comparison with Xpresso [4], histone and Y chromosome genes were discarded, and only protein-coding genes were kept. Ultimately, 18,377 genes were used by Xpresso. Out of these, 18,347 genes also had corresponding expression values in the GDC dataset and were subsequently employed by miREx. The miRNA expression values data downloaded from GDC comprises 1881 miRNAs. Of those, only 243 had known gene targets in the TargetScan database and were used in our model. The CWCS was extracted for each remaining miRNA-target pair from TargetScan.

We computed the mean expression levels of each gene and miRNA across all cancer primary sites. In cases where multiple subtypes were present within a primary site, we also calculated the overall mean expression across all subtypes for that site. The raw counts of mRNA and miRNA were log-normalized $x \leftarrow log_{10}(x+0.1)$ to reduce the right skew of the data.

### Method

**Fig. 1:**
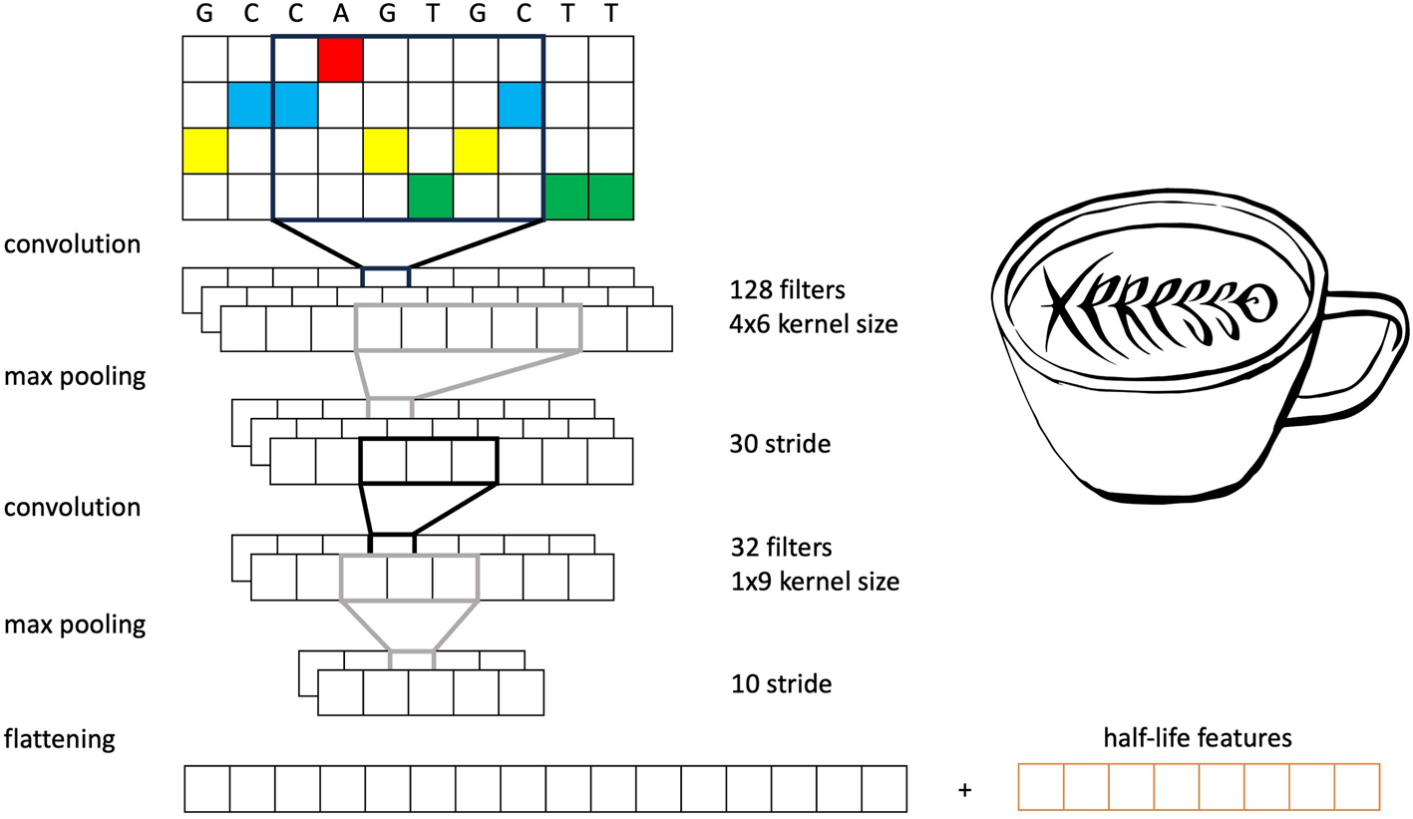
The encoded input sequences are processed by the Xpresso backbone architecture and concatenated to the half-life features [4]

**Fig. 2:**
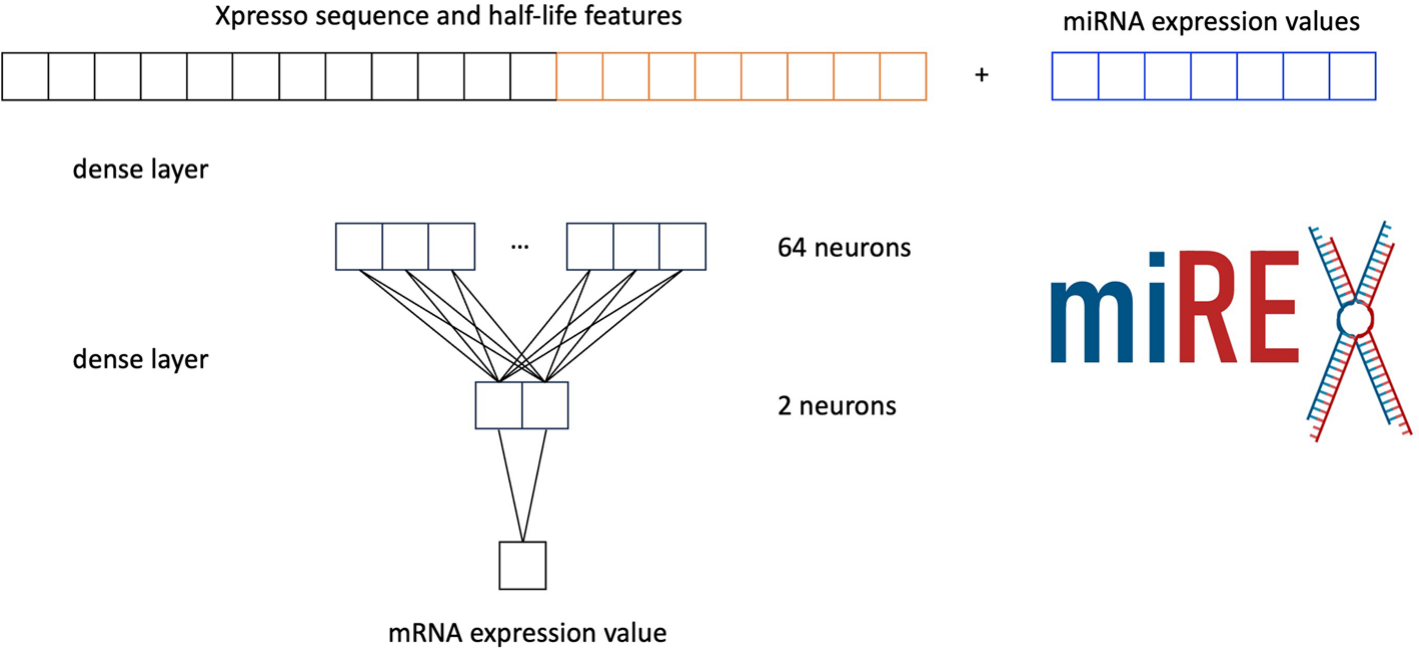
MiREx incorporates miRNA expression and targeting information by concatenating it with sequence and gene features

MiREx exploits the Xpresso CNN architecture as a backbone. It consists of convolutional and max-pooling layers applied on the one-hot encoded DNA sequence. The max pooling output is flattened and concatenated to the previously described eight half-life features. A complete figure describing the Xpresso backbone is reported in Fig. 1. In our model, miRNA expression levels are also concatenated to the DNA sequence and half-life features, as shown in Fig. 2. Finally, two densely connected layers output the results.

Post-transcriptional information is exploited, providing gene-specific miRNA expression levels after the last max pooling layer as a vector. Each element of the vector *x* encodes miRNA *i*. *x* is specific for each gene, according to which miRNAs target it. For each gene, $x_i$ is the expression level of miRNA *i* if miRNA *i* targets the gene, 0 otherwise. Table 1 reports an example of *x* vectors for some genes.

**Table 1:** Example of matrix obtained by miRNA targeting

	$miRNA _1$	$miRNA _2$	...	$miRNA _N$
$Gene_1$	0	1.34		0
$Gene_2$	0	0		1.26
...				
$Gene_N$	1.1	0		0

The dataset was partitioned into training, validation, and test sets. For a fair comparison, we employed the same split used by Xpresso (16,348 genes in training, 1000 in validation, and 999 in test). We used an early stopping procedure to avoid overfitting. Indeed, the training was stopped if the loss on the validation set did not improve for 20 epochs. The rectified linear unit (ReLU) was used as the activation function for convolutional and dense layers. Stochastic Gradient Descent (SGD) served as the optimization algorithm. The Mean Square Error (MSE) is minimized during the network training.

Performances were evaluated with the coefficient of determination ($R^2$), which measures how well the model predicts the ground truth. Given the vector with ground truth expression levels *y*, their mean value $\bar{y}$, and the vector with the predicted expression levels $\hat{y}$, the Residual Sum of Squares and the Total Sum of Squares are defined as follows:

$$\begin{aligned} RSS= & {} \sum _{i=1}^n(y_i - \hat{y_i})^2 \nonumber \\ TSS= & {} \sum _{i=1}^n(y_i - \bar{y})^2 \end{aligned}$$

Finally, the value of $R^2$ is computed as:

$$\begin{aligned} R^2 = 1- \frac{RSS}{TSS} \end{aligned}$$

The parameters used for the training of the model are shown in Table 2.

**Table 2:** Parameter configuration values

Parameter name	Parameter value
Batch size	32
Learning rate	0.0005
Number of epochs	100
Early stopping patience	20

**Xpresso** does not consider the effect of regulatory molecules. Therefore, upregulated genes (e.g., genes regulated by enhancers that increase the transcription rate) should have positive residuals, and downregulated genes (e.g., genes targeted by miRNAs that repress transcription) should have negative residuals. Where residuals are computed as $mRNA\ ground\ truth\ value -mRNA\ predicted\ value$.

We train a model using all miRNAs for which we have both expression levels and miRNA targeting information. In the results section, we call this model **AllMirna**. This method considers targeting information but not the CWCS information from TargetScan.

A gene targeted by a miRNA can have negative residuals that depend on the miRNA’s effectiveness at repressing transcription. TargetScan provides the CWCS, a measure of the likelihood of miRNA binding for each target gene. The lower the CWCS, the greater the probability that the miRNA will bind to the mRNA, repressing its transcription. Consequently, there should be a positive correlation between CWCS and residuals (the more negative the residual, the more negative the CWCS). Therefore, for each miRNA, we computed the Spearman correlation between the residuals and the CWCS, and the ten miRNAs with the highest correlation were chosen. Our model, **miREx**, uses target information from those ten miRNAs.

Although miRNAs can directly impact translation, lowering mRNA expression, indirect regulations could lead to the opposite effect. We also considered the correlation between the absolute values of the residuals and the CWCS. The ten miRNAs with the highest correlation were selected to train another model. In the results section, we call this model **AbsCorr**.

In the end, four different models were trained:

- proposed model: **miREx**,
- sota competitor: **Xpresso**,
- additional configuration: **AllMirna**,
- additional configuration: **AbsCorr**.

The models were trained for each primary site and cancer subtype. Multiple runs were executed, and the best ten were considered for the results.

## Results

In this section, we present the results obtained from the four datasets.

As previously outlined in Sect. Materials and methods, our approach involves training four distinct models. miREx, our proposed model, leverages multiple aspects of miRNAs (expression levels, targeting, and CWCS) as well as sequence and gene features. In contrast, the state-of-the-art model (Xpresso) only uses sequence and gene features. AllMirna includes expression levels and targeting of miRNA but does not take into account the CWCS. Finally, AbsCorr, similarly to miREx, considers all available information for miRNAs; however, AbsCorr examines indirect regulation.

In all the plots in the results section, we display the mean of the $R^2$ values along with their corresponding 95% confidence intervals.

### Lung

**Fig. 3:**
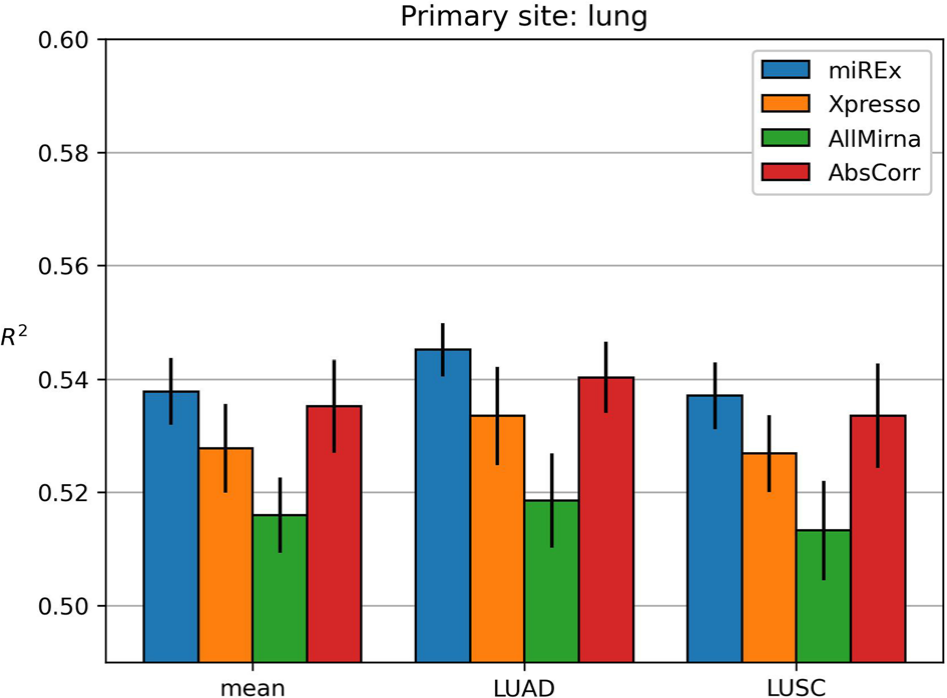
Results obtained on lung dataset

Figure 3 shows the results obtained on the lung dataset. MiREx and the other models were trained with three targets: LUAD, LUSC, and their mean.

Firstly, we compared the Xpresso results with those obtained with the AllMirna model. The $R^2$ decreased with the inclusion of miRNA expression levels in all three cases (LUAD, LUSC, and mean). The differences are statistically significant, with p-values < 0.05. MiREx and AbsCorr were trained using only a subset of the miRNAs, and while both approaches lead to higher $R^2$ in comparison to Xpresso, statistical tests show that only miREx’s results are statistically significant (p-values < 0.05).

Table 3b contains the subsets of ten miRNAs selected by the two methods in the three categories mean, LUAD and LUSC.

There is a significant overlap in the miRNAs chosen by the models in the three categories. Table 3 shows that, with miREx, seven miRNAs are common in the three categories, and two are common between mean and LUSC. Table 3a shows the miRNAs chosen by AbsCorr. Four miRNAs are common, and five are in two categories: mean and LUAD.

There are almost no miRNAs in common between the two methods. mir-23a and mir-23b are the only two in common.

**Table 3:** Lists of miRNAs chosen for each cancer subtype of the lung site by miREx and AbsCorr methods

(a) miREx			(b) AbsCorr		
Mean	LUAD	LUSC	Mean	LUAD	LUSC
**mir-23a**	**mir-23a**	**mir-23a**	**mir-15a**	**mir-15a**	**mir-15a**
**mir-23b**	**mir-23b**	**mir-23b**	**mir-15b**	**mir-15b**	**mir-15b**
**mir-101-2**	**mir-101-2**	**mir-101-2**	**mir-200c**	**mir-200c**	**mir-200c**
**mir-145**	**mir-145**	**mir-145**	**mir-340**	**mir-340**	**mir-340**
**mir-199b**	**mir-199b**	**mir-199b**	**mir-23b**	**mir-23b**	mir-130a
**mir-206**	**mir-206**	**mir-206**	**mir-195**	**mir-195**	mir-130b
**mir-655**	**mir-655**	**mir-655**	**mir-424**	**mir-424**	mir-301b
**mir-19a**	mir-20b	**mir-19a**	**mir-429**	**mir-429**	mir-301a
**mir-506**	mir-93	**mir-506**	**mir-497**	**mir-497**	mir-454
mir-140	mir-101-1	mir-381	mir-200b	mir-23a	mir-506

Common miRNAs are in bold text

### Kidney

**Fig. 4:**
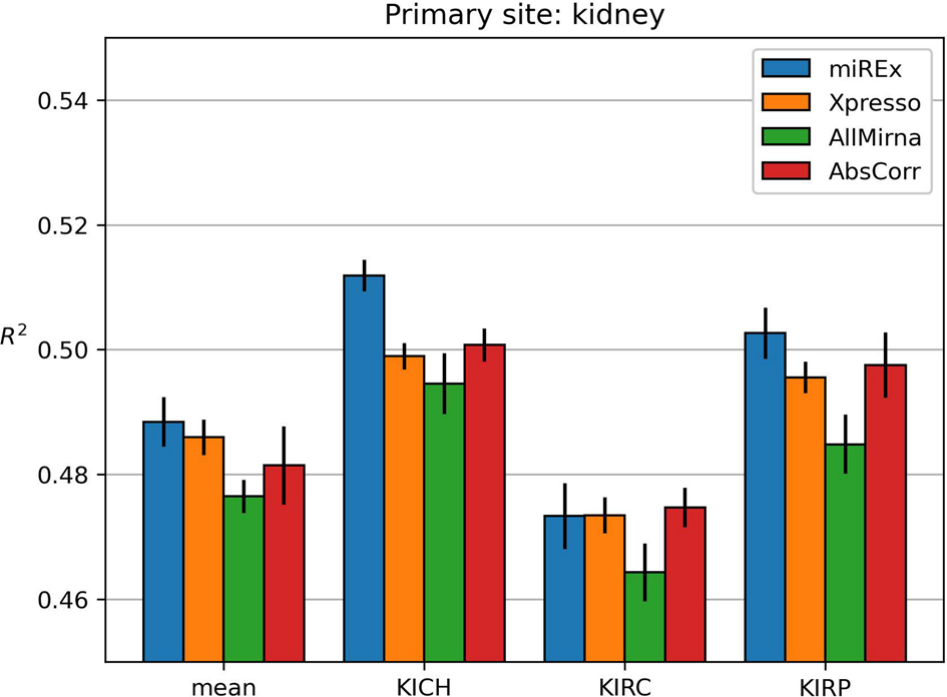
Results obtained on kidney dataset

Figure 4 shows the results obtained on the kidney dataset. The models were trained with four targets: KICH, KIRC, KIRP, and the mean of all three classes.

We can see that in all four cases, the AllMirna model leads to worse results. In two classes (KICH and KIRP), we can see that using a selected subset of miRNAs leads to better predictions when compared to Xpresso. In particular, with miREx, the results are statistically significant (p-value $<<$ 0.05). The third class, KIRC, has a different trend. In this case, our model does not seem to bring an improvement in the results. The results on the mean of all three subtypes show an improvement with our model. However, it is not statistically significant.

Table 4b displays the miRNAs selected by MiREx and AbsCorr. Again, there is a high overlap between miRNAs chosen by the same method in the different classes, while there is a low overlap between the two techniques.

**Table 4:** Lists of miRNAs chosen for each cancer subtype of the kidney site by miREx and AbsCorr methods

miREx				AbsCorr			
**KICH**	**KIRP**	**KIRC**	**Mean**	**KICH**	**KIRP**	**KIRC**	**Mean**
**mir-1**	**mir-1**	**mir-1**	**mir-1**	**mir-124-1**	**mir-124-1**	**mir-124-2**	**mir-124-1**
**mir-124-1**	**mir-124-1**	**mir-124-1**	**mir-124-1**	**mir-30a**	**mir-30a**	**mir-30a**	**mir-30a**
**mir-124-2**	**mir-124-2**	**mir-124-2**	**mir-124-2**	**mir-30b**	**mir-30b**	**mir-30b**	**mir-30b**
**mir-142-1**	**mir-142-1**	**mir-142-1**	**mir-142-1**	**mir-30c**	**mir-30c**	**mir-30c**	**mir-30c**
**mir-142-2**	**mir-142-2**	**mir-142-2**	**mir-142-2**	**mir-30d**	**mir-30d**	**mir-30d**	**mir-30d**
**mir-145**	**mir-145**	**mir-145**	**mir-145**	**mir-340**	**mir-340**	**mir-340**	**mir-340**
**mir-199a**	**mir-199a**	**mir-199a**	**mir-199a**	**mir-429**	**mir-429**	mir-506	**mir-429**
**mir-206**	**mir-206**	**mir-206**	**mir-206**	**mir-30e**	**mir-30e**	mir-23c	let-7i
**mir-506**	**mir-506**	**mir-506**	**mir-506**	**mir-200b**	**mir-200b**	mir-23a	let-7g
**mir-199b**	mir-325	**mir-199b**	**mir-199b**	**mir-200c**	**mir-200c**	mir-23b	let-7b

Common miRNAs are in bold text

### Breast

**Fig. 5:**
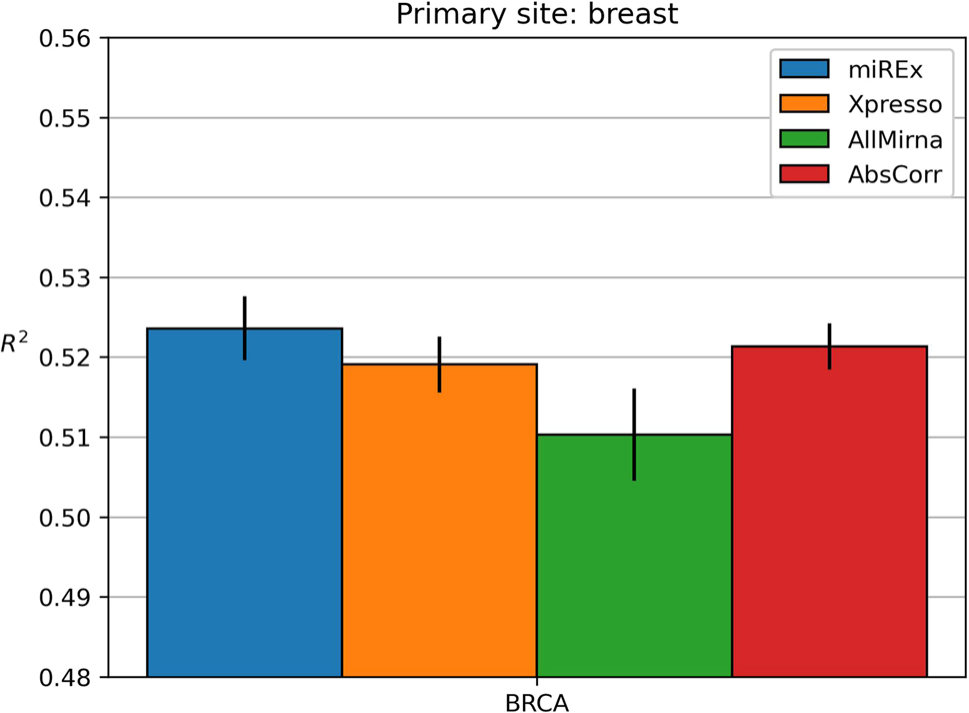
Results obtained on breast dataset

Figure 5 shows the results obtained on the breast dataset. The models were trained with a single breast cancer subtype: BRCA.

The results are similar to those obtained with the other datasets: including all miRNAs leads to worse predictions than Xpresso (p-value < 0.05) while adding a subset of miRNAs leads to better results, where miREx is the best model. While it is true that the models using a subset of miRNAs have higher mean $R^2$, in this case, both miREx and AbsCorr have p-values > 0.05, with miREx coming close (0.06) but not enough to be considered statistically significant.

Table 5b shows the miRNA selected by miREx and AbsCorr. The two methods show a limited overlap of miRNAs, as only three are in common.

**Table 5:** Lists of miRNAs chosen for the BRCA cancer subtype of the breast site by miREx and AbsCorr methods

(a) miREx	(b) AbsCorr
BRCA	BRCA
mir-1	mir-124-1
mir-124-1	mir-124-2
mir-124-2	mir-15a
mir-142-1	mir-15b
mir-142-2	mir-16
mir-199a	mir-195
mir-199b	mir-200b
mir-206	mir-340
mir-325	mir-497
mir-506	mir-506

### Corpus Uteri

**Fig. 6:**
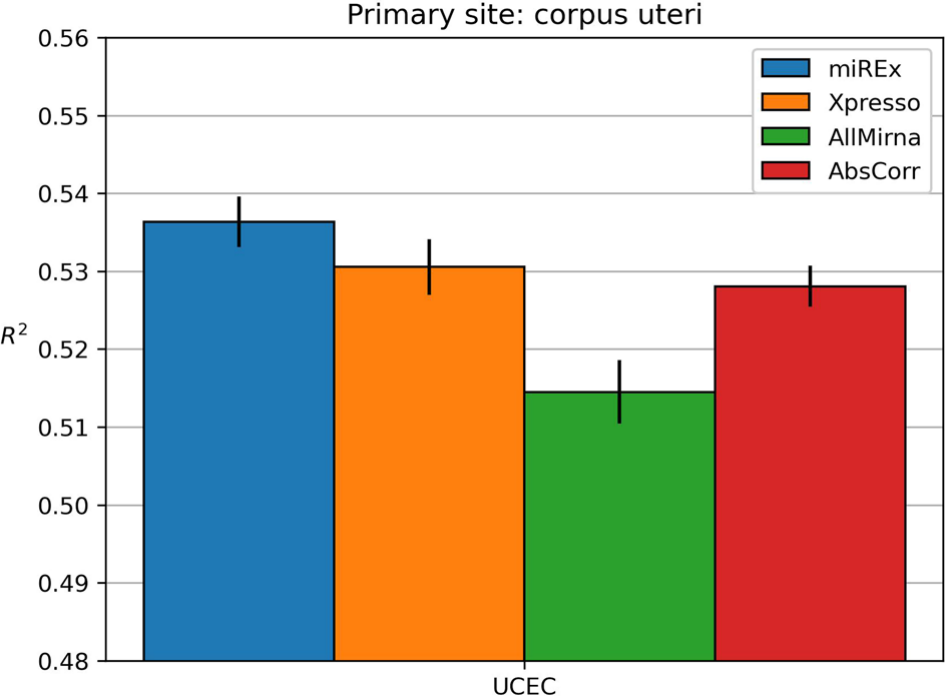
Results obtained on corpus uteri dataset

Figure 6 shows the results obtained on the corpus uteri dataset. The models were trained with a single corpus uteri cancer subtype: UCEC.

In this dataset, AllMirna and AbsCorr lead to worse results than Xpresso. MiREx improves the prediction (p-value < 0.05).

Table 6b displays the miRNAs chosen by miREx and AbsCorr. The overlap of miRNAs is minimal, as only two are in common.

**Table 6:** Lists of miRNAs chosen for the UCEC cancer subtype of the corpus uteri site by miREx and AbsCorr methods

(a) miREx	(b) AbsCorr
UCEC	UCEC
mir-1	mir-124-1
mir-124-1	mir-15a
mir-124-2	mir-15b
mir-142-1	mir-16
mir-142-2	mir-195
mir-145	mir-200b
mir-199a	mir-200c
mir-206	mir-340
mir-325	mir-424
mir-506	mir-506

## Discussion

As shown in the results Sect. Data description, training the model using all the miRNAs led to lower $R^2$ values. Most genes are not targeted by a high number of miRNAs. Because of that, the data in the vectors created for each gene was very sparse. It is possible that the model could not extract the information effectively.

To solve this problem, we propose miREx, a model that only uses a subset of miRNAs with a high impact on transcriptional regulation. Since the regulation of miRNAs can be indirect, a second model that considers indirect regulatory mechanisms was trained. Both models lead to higher $R^2$, but the results show that direct regulation has a greater effect. Indeed, the miREx model is the one with the best performance.

We examined the miRNAs selected for each model to see if they regulate specific genes and explain why they have a more significant impact. As expected, the two methods chose different miRNAs since they looked at miRNAs with direct or indirect effects on gene regulation. Instead, when there are multiple classes for the same cancer primary site, the miRNAs chosen by the same method in the different categories are very similar.

Many of the miRNAs selected by these methods are known to play various roles in cancer-related processes. For example, miR-23a is involved in many cancers [29], and, in particular, in non-small cell lung cancer (NSCLC) [30, 31], which is a class of lung cancers that includes LUAD and LUSC, two subtypes used in this work. At least one method chose miR-506 for all four datasets. This miRNA is known for its association with different cancers, including kidney [32] and lung [33].

## Conclusions

This work aimed to build a model that considers the post-transcriptional regulation of miRNAs to predict mRNA levels. MiREx, the model proposed in this paper, is a CNN that takes as input the one-hot encoded sequence of a portion of a gene, mRNA half-life features, and includes information about miRNA expression levels and targeting to predict mRNA expression levels.

Future works might use these methods to predict protein expression levels. MiRNAs should have a more significant impact on protein expression levels since multiple regulation mechanisms would be considered. With mRNAs, the regulatory mechanism under consideration is the one where the miRNAs cause the degradation of the mRNA molecule so that it will not be translated. For proteins, we expect to be able to see the effects of miRNAs on translational inhibition of mRNA without causing its degradation.

## Data Availability

The code underlying this article is available in the GitHub repository.

## Availability of data and materials

The datasets analyzed during the current study are available in the Xpresso repository [4]. The datasets generated during the current study are available in the miREx zenodo repository.
